# Supplementary material for: Centriole growth is limited by the Cdk/Cyclin-dependent phosphorylation of Ana2/STIL
Source: J Cell Biol. 2022 Jul 21;221(9):e202205058. doi: 10.1083/jcb.202205058 (PMC9442473; doi:10.1083/jcb.202205058)
Supplement: Table S1 — shows alleles and fly stocks used in this study [file JCB_202205058_TableS1.docx]

Table S1: Alleles and fly stocks used in this study

| Alleles used in this study | Source |
| --- | --- |
| p(Sas-6)-mNG | (Alvarez-Rodrigo et al., 2019) |
| p(Sas-6)-dNG | (Alvarez-Rodrigo et al., 2019) |
| eAsl-mKate2 | (Aydogan et al., 2020) |
| asl^B46^ | (Baumbach et al., 2015) |
| mNG-Asl | This paper ; CRISPR/Cas9 knock-in |
| Asl-mNG | This paper ; CRISPR/Cas9 knock-in |
| mNG-Sas-6 | This paper ; CRISPR/Cas9 knock-in |
| Sas-6-mNG | This paper ; CRISPR/Cas9 knock-in |
| Sas-4-mNG | This paper ; CRISPR/Cas9 knock-in |
| mNG-Ana2 | This paper ; CRISPR/Cas9 knock-in |
| Ana2-mNG | This paper ; CRISPR/Cas9 knock-in |
| mNG-Plk4 | This paper ; CRISPR/Cas9 knock-in |
| ePlk4-mNG | (Aydogan et al., 2020) |
| Plk4^Aa74^ | (Aydogan et al., 2018) |
| eSas-6-GFP | (Aydogan et al., 2018) |
| Sas-6^c02901^ | (Peel et al., 2007) |
| ana2^Δa^ | This paper ; CRISPR/Cas9 knock-out |
| ana2^Δb^ | This paper ; CRISPR/Cas9 knock-out |
| WT eAna2-mNG | This paper ; transgenic allele expressed from core promoter |
| eAna2(ΔCC)-mNG#4 | This paper ; transgenic allele expressed from core promoter |
| eAna2(ΔSTAN)-mNG#2 | This paper ; transgenic allele expressed from core promoter |
| eAna2(12A)-mNG | This paper ; transgenic allele expressed from core promoter |
| eAna2(12A) | This paper ; transgenic allele expressed from core promoter |
| eAna2(12D/E)-mNG | This paper ; transgenic allele expressed from core promoter |
| Fly stocks used in this study | **Figure** |
| Oregon-R (Wild type control) | 1, 6, S1, S5C,D |
| w^67^ (used as WT control in some experiments) | S5A,B |
| w;;p(Sas-6)-mNG/eAsl-mKate2, asl^B46^ | 2, 3, S2 |
| w;;p(Sas-6)-dNG/eAsl-mKate2, asl^B46^ | 2, 3, S2 |
| w;; mNG-Asl/+ | 1 |
| w;; Asl-mNG/Asl-mNG | 1, 2, 3, S2 |
| w;; Asl-mNG/+ | 1 |
| w;; mNG-Sas-6/mNG-Sas-6 | 1, 2, 3, S2 |
| w;; mNG-Sas-6/+ | 1 |
| w;; Sas-6-mNG/Sas-6-mNG | 1, 2, 3, S2 |
| w;; Sas-6-mNG/+ | 6, 1 |
| w;; Sas-4-mNG/Sas-4-mNG | 1, 2, 3, S2 |
| w;; Sas-4-mNG/+ | 1 |
| w; mNG-Ana2/mNG-Ana2 | 1, 2, 3, S2 |
| w; mNG-Ana2/+ | 1 |
| w; Ana2-mNG/Ana2-mNG | 1, 2, 3, 5, S1, S2, S5 |
| w; Ana2-mNG/+ | 1, 8, S1, S5 |
| w;; mNG-Plk4/mNG-Plk4 | 1 |
| w;; ePlk4-mNG, Plk4^Aa74^/ ePlk4‑mNG, Plk4^Aa74^ | 1, 2, 7 |
| w; eSas-6-GFP#1/+ ; Sas-6^c02901^/Sas-6^c02901^ | S1 |
| w; eSas-6-GFP#1/eSas-6-GFP#1 ; Sas-6^c02901^/Sas-6^c02901^ | S1 |
| w; eSas-6-GFP#1/+ ; Sas-6^c02901^, eSas-6-GFP#2/+ | S1 |
| w; eSas-6-GFP#1/eSas-6-GFP#1 ; Sas-6^c02901^, eSas-6-GFP#2/Sas‑6^c02901^, eSas-6-GFP#2 | S1 |
| w; ana2^Δa^/+ ; eAna2‑mNG/ eAsl-mKate2, asl^B46^ | 4, S1 |
| w; ana2^Δa^/+ ; eAna2(ΔCC)-mNG/eAsl-mKate2, asl^B46^ | 4, S1 |
| w; ana2^Δa^/+ ; eAna2(ΔSTAN)-mNG/eAsl-mKate2, asl^B46^ | 4, S1 |
| w; ana2^Δa^/ana2^Δa^ ; eAna2-mNG/eAna2-mNG | 4, S1 |
| w; ana2^Δb^, eAna2(12A)-mNG/ana2^Δb^, eAna2(12A)-mNG | 4, 5, S5 |
| w; ana2^Δb^, eAna2(12A)-mNG/+ | S5 |
| w; ana2^Δa^, eAna2(12A)/ ana2^Δa^, eAna2(12A) | S5 |
| w; ana2^Δa^, eAna2(12A)/ana2^Δa^, eAna2(12A) ; Sas-6-mNG/+ | 6 |
| w; ana2^Δa^, eAna2(12A)/+ ; ePlk4-mNG, Plk4^Aa74^/ ePlk4‑mNG, Plk4^Aa74^ | 7 |
| w; ana2^Δa^, eAna2(12D/E)-mNG/+ | 8, S5 |
| w; ana2^Δa^, eAna2(12D/E)-mNG/ ana2^Δa^, eAna2(12D/E)-mNG | S5 |
| w; Ana2-mNG/ana2^Δa^ | S1 |

**References:**

Alvarez-Rodrigo, I., T.L. Steinacker, S. Saurya, P.T. Conduit, J. Baumbach, Z.A. Novak, M.G. Aydogan, A. Wainman, and J.W. Raff. 2019. Evidence that a positive feedback loop drives centrosome maturation in fly embryos. *eLife*. 8:1–31. doi:10.7554/eLife.50130.

Aydogan, M.G., T.L. Steinacker, M. Mofatteh, Z.M. Wilmott, F.Y. Zhou, L. Gartenmann, A. Wainman, S. Saurya, Z.A. Novak, S.-S. Wong, A. Goriely, M.A. Boemo, and J.W. Raff. 2020. An Autonomous Oscillation Times and Executes Centriole Biogenesis. *Cell*. 181:1566-1581.e27. doi:10.1016/j.cell.2020.05.018.

Aydogan, M.G., A. Wainman, S. Saurya, T.L. Steinacker, A. Caballe, Z.A. Novak, J. Baumbach, N. Muschalik, and J.W. Raff. 2018. A homeostatic clock sets daughter centriole size in flies. *Journal of Cell Biology*. 217:1233–1248. doi:10.1083/jcb.201801014.

Baumbach, J., Z.A. Novak, J.W. Raff, and A. Wainman. 2015. Dissecting the Function and Assembly of Acentriolar Microtubule Organizing Centers in Drosophila Cells In Vivo. *PLoS Genetics*. 11:1–22. doi:10.1371/journal.pgen.1005261.

Peel, N., N.R. Stevens, R. Basto, and J.W. Raff. 2007. Overexpressing Centriole-Replication Proteins In Vivo Induces Centriole Overduplication and De Novo Formation. *Current Biology*. 17:834–843. doi:10.1016/j.cub.2007.04.036.
