# Supplementary material for: Centriole growth is limited by the Cdk/Cyclin-dependent phosphorylation of Ana2/STIL
Source: J Cell Biol. 2022 Jul 21;221(9):e202205058. doi: 10.1083/jcb.202205058 (PMC9442473; doi:10.1083/jcb.202205058)
Supplement: Table S2 — shows primers and gRNA sequences used in this study [file JCB_202205058_TableS2.docx]

Table S2: Primers and gRNA sequences used in this study

| Aim | Primer sequences (5’-) |
| --- | --- |
| Insertion of Sas-6 N-terminal gRNA sequence into the pCFD3 plasmid (KI) | GTCG**AGTAGCTATCCTCGCTCCC**  AAAC**GGGAGCGAGGATAGCTACT** |
| Insertion of Sas-6 C-terminal gRNA sequence into the pCFD3 plasmid (KI) | GTCG**AGAACGGCTTGCAATACCCA**  AAAC**TGGGTATTGCAAGCCGTTCT** |
| Insertion of Ana2 N-terminal gRNA sequence into the pCFD3 plasmid (KI) | GTCG**GCAGCATATCCTCCGTTTC**  AAA**CGAAACGGAGGATATGCTGC** |
| Insertion of Ana2 C-terminal gRNA sequence into the pCFD3 plasmid (KI) | GTCG**CTTTCACAACAGCTTCGGC**  AAAC**GCCGAAGCTGTTGTGAAAG** |
| Insertion of Asl N-terminal gRNA sequence into the pCFD3 plasmid (KI) | GTCG**ATACCTGGCGTGTTCATATT**  AAAC**AATATGAACACGCCAGGTAT** |
| Insertion of Asl C-terminal gRNA sequence into the pCFD3 plasmid (KI) | GTCG**TTAGCTGTGACCATTGCCTT**  AAAC**AAGGCAATGGTCACAGCTAA** |
| Insertion of Sas-4 C-terminal gRNA sequence into the pCFD3 plasmid (KI) | GTCG**AGAGACCCGACTCTAATACT**  AAAC**AGTATTAGAGTCGGGTCTCT** |
| Insertion of Plk4 N-terminal gRNA sequence into the pCFD3 plasmid (KI) | GTCG**CTAGCTATGTTATCCAATC**  AAAC**GATTGGATAACATAGCTAG** |
| Primers for the NEBuilder assembly of the Sas-6 N-terminal donor plasmid | GGGAGCGAGGATAGCTACTCGAATTCGATATCAAGCTTATCGATA  GGGAGCGAGGATAGCTACTCACTAGTTCTAGAGCGGCCGCCA  GAGTAGCTATCCTCGCTCCCTGGGTGGTCCACTGTTGTCCCGCTG  TGCTCACCATGATAGATCAGGCTCGTGAGAAAC  CTGATCTATCATGGTGAGCAAGGGCGAGGAGG  ACTATACGAGTCTTCTGATCCTGGAGGCCACATGAAGCCTGCTTTTTTGTACAAACT  GATCAGAAGACTCGTATAGTGCCAAAATGGACTATGGCAAGAG  GAGTAGCTATCCTCGCTCCCTGGCAGTGTGCTGCTTGAGGTCCTC |
| Primers for the NEBuilder assembly of the Sas-6 C-terminal donor plasmid | TGGGTATTGCAAGCCGTTCTGAATTCGATATCAAGCTTATCGATA  TGGGTATTGCAAGCCGTTCTACTAGTTCTAGAGCGGCCGCCA  AGAACGGCTTGCAATACCCACGGCAACGCACAACGCGCGCATGCG  TAGGGATGGCCAATCTCTCCTTTAGAATGAGGATTTCTGAAAAGAG  GGAGAGATTGGCCATCCCTACTGGCAAAGAAAATCGGCGAGACCCAGCTTTCTTGTACAAAGTG  ATAAGTAAAATTACTTGTACAGCTCGTCCATGC  GTACAAGTAATTTTACTTATTGTTAATGCATTTTTTC  AGAACGGCTTGCAATACCCACGGCGCTGTGTATCCATCTTGGCCGC |
| Primers for the NEBuilder assembly of the Ana2 N-terminal donor plasmid | GAAACGGAGGATATGCTGCCGAATTCGATATCAAGCTTATCGATA  GAAACGGAGGATATGCTGCCACTAGTTCTAGAGCGGCCGCCA  GGCAGCATATCCTCCGTTTCGGGCCGTGAAATGCCCAGCGAGCTG  TGCTCACCATTTGGAGCGTATTTGTTTATATTTGC  TACGCTCCAAATGGTGAGCAAGGGCGAGGAGG  GGTAACATGTCTTCAGTCTCGGGAACAAACATGAAGCCTGCTTTTTTGTACAAACT  GAGACTGAAGACATGTTACCCAGACTAGCGCCCAGGCCGAGT  GGCAGCATATCCTCCGTTTCGGGGCAAGTATTTCAGCGCCAGCTCG |
| Primers for the NEBuilder assembly of the Ana2 C-terminal donor plasmid | GCCGAAGCTGTTGTGAAAGCGAATTCGATATCAAGCTTATCGATA  GCCGAAGCTGTTGTGAAAGCACTAGTTCTAGAGCGGCCGCCA  GCTTTCACAACAGCTTCGGCTGGGGCCACAGAACTGGGTCCTCGC  TAGTAATTTAGGTTGATTCCTGATATTCTCCAAGTCCAGT  GGAATCAACCTAAATTACTAGACCCAGCTTTCTTGTACAAAGTG  GTACATGCTTTTACTTGTACAGCTCGTCCATGC  GTACAAGTAAAAGCATGTACAATGTTCGTTTTGTT  GCTTTCACAACAGCTTCGGCTGGGGACCCCTCTCAATATCAGGTGG |
| Primers for the NEBuilder assembly of the Asl N-terminal donor plasmid | CCGAATATGAACACGCCAGGTATACTAGTTCTAGAGCGGCCGCCA  CCGAATATGAACACGCCAGGTATGAATTCGATATCAAGCTTATCGATA  CCTGGCGTGTTCATATTCGGCCAAGGTTTTCCAAATAGATCCCG  TGCTCACCATATTCAGCTAAGGGGACGCCACAAGCAT  TTAGCTGAATATGGTGAGCAAGGGCGAGGAGG  TTATCCCGGGAGTATTCATGAAGCCTGCTTTTTTGTACAAACT  CATGAATACTCCCGGGATAAGCCTCTTTCAGGGGGCGGA  CCTGGCGTGTTCATATTCGGCAATGCGGGCTTTCAAGTCAACG |
| Primers for the NEBuilder assembly of the Asl C-terminal donor plasmid | CCAAAGGCAATGGTCACAGCTAAACTAGTTCTAGAGCGGCCGCCA  CCAAAGGCAATGGTCACAGCTAAGAATTCGATATCAAGCTTATCGATA  GCTGTGACCATTGCCTTTGGGGAAATTCAGTTGTTGAAACTCCA  AGAATGGCCGTTGCCCTTAGGCTTTCTATTTGAGTTGGTGATTG  CTAAGGGCAACGGCCATTCTGACCCAGCTTTCTTGTACAAAGTG  TTCCTAAGTCTTACTTGTACAGCTCGTCCATGC  GTACAAGTAAGACTTAGGAAAATATATATATGTATAT  GCTGTGACCATTGCCTTTGGGAGTGGATAGCATCCTCTGCCTG |
| Primers for the NEBuilder assembly of the Sas-4 C-terminal donor plasmid | CCAAGTATTAGAGTCGGGTCTCTACTAGTTCTAGAGCGGCCGCCA  CCAAGTATTAGAGTCGGGTCTCTGAATTCGATATCAAGCTTATCGATA  GACCCGACTCTAATACTTGGCAGTGAGAAGGAGCAGCGAAACT  TGGGTCGTATTTAGCATAGTCTGTGTCCATTATGAGCT  ACTATGCTAAATACGACCCAGCTTTCTTGTACAAAGTG  AGACCCGACTTTACTTGTACAGCTCGTCCATGC  GTACAAGTAAAGTCGGGTCTCTGCTTCCGTTG  GACCCGACTCTAATACTTGGTGTTCGCTGCATTCTTGTTGAGTT |
| Primers for the NEBuilder assembly of the Plk4 N-terminal donor plasmid | CCCGATTGGATAACATAGCTAGCACTAGTTCTAGAGCGGCCGCCA  CCCGATTGGATAACATAGCTAGCGAATTCGATATCAAGCTTATCGATA  AGCTATGTTATCCAATCGGGGTGTGAGAGTCCAAGGTTGTCTG  TGCTCACCATAGCTAGCCTTTTTTCTGTAGACTTAC  AAGGCTAGCTATGGTGAGCAAGGGCGAGGAGG  AACGCTCTGTTACTGAGCATGAAGCCTGCTTTTTTGTACAAACTT  ATGCTCAGTAACAGAGCGTTTGGAGAAACAATTGAGGTG  AGCTATGTTATCCAATCGGGTACTGCTAGCAAATGTTATGATTCC |
| Screening primers inside mNG | CCCGTCAGGGTAGGGCAGGTAC  GAAGACCGAGCTGAAGCACTCCA |
| Screening primers for mNG-Sas-6 | CTCCCCTATATCCGCTGGTTGGA  CACATACCTTCTCTTTGTTTCCCT |
| Screening primers for Sas-6-mNG | CAGCATGCTGGAAGCCTCCCAC  CAGCAGATTTCCGATTTCCACCC |
| Screening primers for mNG-Ana2 | CGCCGAGGAAGAGCTGCAGCTG  CGCCCCCAGGCGCATATCCTTC |
| Screening primers for Ana2-mNG | CCTCGTGCTGCACCCACCTTCG  CCATCCCCTGTTCCCAGTCGAC |
| Screening primers for mNG-Asl | CTTTGATGCGCAAAGTTGGAAACG  CGAAGCGACTGTTTGCTCCAAATA |
| Screening primers for Asl-mNG | GCGATAACCTTTCAGACATGCTAG  GGAGAGTCCCTGAACACGAACGT |
| Screening primers for Sas-4-mNG | GCAGGCGCATGTCTCGGCACAG  CTCTGATCTGGCAACGCCAGGC |
| Screening primers for mNG-Plk4 | TCATTGACGTGTGTGAGAGTCCAA  CAAATGTACATTGTAAATTCCTGAAT |
| Primers for the insertion of the ana2 gRNA sequence into the pCFD4 plasmid (KO); for generation of ana2^Δa^ | TATATAGGAAAGATATCCGGGTGAACTTC**GGCAGCATATCCTCCGTTTC**GTTTTAGAGCTAGAAATAGCAAG  ATTTTAACTTGCTATTTCTAGCTCTAAAAC**AAGCTGTTGTGAAAGCATGTC**GACGTTAAATTGAAAATAGGTC |
| Primers for the insertion of the ana2 gRNA sequence into the pCFD4 plasmid (KO); for generation of ana2^Δb^ | TATATAGGAAAGATATCCGGGTGAACTTC**GCCGTTTCGGGAACAAACATT**GTTTTAGAGCTAGAAATAGCAAG  ATTTTAACTTGCTATTTCTAGCTCTAAAAC**GCCGAAGCTGTTGTGAAAGC**GACGTTAAATTGAAAATAGGTC |
| Primers for the screening of the ana2 knock-out deletions | CTGTTCTCAGCTGGAGTCGGAGTCTCTGC  TCGCCTTCGGAACGGACTTTGCGCAGTGC |
| Primers for the amplification of the DEST vector containing the ana2 promoter and C-terminal mNG | GAAGCTGTTGGACCCAGCTTTCTTGTACAAAGTG  GAACAAACATTTGGAGCGTATTTGTTTATATTTGCC |
| Primers for the amplification of the ana2 gene and its mutant forms without Stop codon | TACGCTCCAAATGTTTGTTCCCGAAACGGAGGAT  AAGCTGGGTCCAACAGCTTCGGCTGGTTCCTGA |
| Primers for the amplification of the DEST vector containing the ana2 promoter | GCTGTTGTGACCGCGGTGGCGGCCGTCTAGAA  GAACAAACATTTGGAGCGTATTTGTTTATATTTGCC |
| Primers for the amplification of the ana2 gene and its mutant forms with Stop codon | TACGCTCCAAATGTTTGTTCCCGAAACGGAGGAT  GCCACCGCGGTCACAACAGCTTCGGCTGGTTCC |
| Primers for the reintroduction of ana2’s intron | TGTATGAAAAGAATCCAATAAAACATCCTTCCTAGCAGGTAGACGCTTGTCTCCCATT  ATTGGATTCTTTTCATACAGATTCAACGTACGCACCTTGATTGGTGGTCAGAATATCGC |
