# Supplementary material for: Centriole growth is limited by the Cdk/Cyclin-dependent phosphorylation of Ana2/STIL
Source: J Cell Biol. 2022 Jul 21;221(9):e202205058. doi: 10.1083/jcb.202205058 (PMC9442473; doi:10.1083/jcb.202205058)
Supplement: Table S3 — shows selected model and anomalous subdiffusion parameter α for all proteins measured with FCS [file JCB_202205058_TableS3.docx]

Table S3: Selected model and anomalous subdiffusion parameter α for all proteins measured with FCS

| Protein | Diffusion model | Anomolous subdiffusion parameter |
| --- | --- | --- |
| mNG | 1 diffusing species, 1 dark state of the fluorophore (triplet state) | 0.75 |
| dNG | 1 diffusing species, 1 dark state of the fluorophore (triplet state) | 0.85 |
| Asl-mNG | 1 diffusing species, 2 dark states of the fluorophore | 0.75 |
| mNG-Sas-6 | 1 diffusing species, 2 dark states of the fluorophore | 0.80 |
| Sas-6-mNG | 1 diffusing species, 2 dark states of the fluorophore | 0.80 |
| Sas-4-mNG | 1 diffusing species, 2 dark states of the fluorophore | 0.80 |
| mNG-Ana2 | 1 diffusing species, 2 dark states of the fluorophore | 0.80 |
| Ana2-mNG | 1 diffusing species, 2 dark states of the fluorophore | 0.80 |
| eAna2-mNG/+ | 1 diffusing species, 2 dark states of the fluorophore | 0.80 |
| eAna2(ΔCC)-mNG/+ | 1 diffusing species, 2 dark states of the fluorophore | 0.85 |
| eAna2(ΔSTAN)-mNG/+ | 1 diffusing species, 2 dark states of the fluorophore | 0.85 |
| eAna2-mNG | 1 diffusing species, 2 dark states of the fluorophore | 0.85 |
| eAna2(12A)-mNG | 1 diffusing species, 2 dark states of the fluorophore | 0.80 |
| eSas-6-GFP | 1 diffusing species, 2 dark states of the fluorophore | 0.75 |
